# Dr. Currie on the Affusion of Cold Water

**Published:** 1801-11

**Authors:** J. Currie

**Affiliations:** Liverpool


### Medical and Phyſical Journal.

VOL. VI.] NOVEMBER, 1801. [NO. XXXIII.

### To Dr. BATTY.

DEAR SIR,

IN the month of October, 1797, I ſubmitted to the public a method of treating fever by the affuſion of water on the ſurface of the body,* which has been repeatedly noticed in your Journal; and ſeveral enquiries having been addreſſed to me reſpecting my ſubſequent experience of the ſame remedy, I beg leave to give my anſwer through the medium of your publication, which ſeems to attract the general attention of the medical world.

Into the particulars of the practice detailed in that work it is unneceſſary to enter. Four years of my farther experience ſerve only to confirm that practice, which is now ſupported by the experience of a conſiderable number of reſpectable practitioners, not in this iſland only but in other parts of the world, of which it is my deſign to lay ſome account before the public. As, however, it is uncertain when this deſign may be carried into effect, I ſubmit in the mean time the following narrative to your readers, in the hope that it may encourage others to follow the practice it ſo powefrully recommends, and with a reſpectful requeſt that they may communicate the reſult of their deliberate experience.

Soon after the Cheſhire regiment of militia, which is now quartered in Liverpool, marched into the town, their commanding officer, Major Buckworth, with the liberal ſpirit of his profeſſion, called upon me to mention the extraordinary ſucceſs of the affuſion of cold water in an epidemic fever which had prevailed in the regiment, and to introduce to me the ſurgeon, Mr. Marſhall, from whom I received the following particulars:

---

* See Medical Reports on the Effects of Water, cold and warm, as a Remedy in Fever and other Diſeaſes, whether applied to the Surface of the Body, or uſed internally, &c. By James Currie, M. D. F. R. S. Publiſhed by Cadell and Davies. Second edition, 1798.

### To Dr. CURRIE.

Sir, *Liverpool, July* 20, 1801.

" In the month of May, 1800, the Cheſhire regiment were in barracks at Goſport, when the typhus fever made its appearance among them. It was probably communicated to them while on duty over the French priſoners, among whom it prevailed. The firſt ſymptoms were a dull head-ach, with reſtleſſneſs and ſhivering, pains in the back and all over the body, the tongue, foul with great proſtration of ſtrength. The head-ach became gradually more acute, the heat roſe to 102° and 104°, and in one inſtance to 107°; and in general the reſtleſſneſs increaſed to delirium, particularly in the night. The fever ſpread with rapidity. At firſt we employed the uſual remedies; emetics in the firſt inſtance, antimonials for a day or two afterwards, to keep the ſkin ſoft, then wine, in proportion to the debility, from a pint to a quart and upwards, daily, with nutritious diet, and an opiate conſiſting of 40 or 50 drops of the tincture of opium every night. The bowels were kept open with calomel and rhubarb, and barley water and lemonade were drank at pleaſure. In four caſes I gave three bottles of wine a day. Theſe ſeemed deſperate, but all of them finally recovered. Port wine ſeemed ſcarcely to ſtimulate in theſe inſtances at all; I changed it for Sherry, and this I ſometimes mixed with brandy. In a ſingle month the wine coſt me ten pounds four ſhillings, though I had a great part of it from the meſs, where it was laid in in quantity, and on the loweſt terms. I uſed few bliſters and no bark. This laſt medicine I had given before, in a number of ſimilar caſes, at Wincheſter, without any benefit. Where there was delirium, which was pretty general, the head was ſhaved. This practice I continued for two months; during that time thirty of the men were ſeized with the infection, and in few or no inſtances was the diſeaſe ſtopped by the emetics or antimonial ſudorifics. The fever ran from thirteen to ſeventeen days, and in ſome caſes to three and four weeks. We loſt during the fever one man only, but ſeveral from the effects combining with other cauſes, particularly pulmonic conſumption.

" The contagion continued to ſpread in ſpite of all our endeavours. At length we had twenty-five in the hoſpital together, in the different ſtages of the diſeaſe.

" Finding none of the uſual means ſucceſsful in arreſting the fever, I had recourſe to the affuſion of cold water; this was towards the end of July. The firſt caſe in which I tried it was that of a battalion man, in the ſecond day of the diſeaſe. I ſtripped him naked and threw four or five gallons of ſea water ter over him, dried him, and put him to bed. He felt very comfortable, became drowſy, and ſlept for two hours; when he awoke his ſkin was cool and moiſt, the pulſe nearly natural, and the head-ach in a great meaſure gone. In the evening the fever returned; I threw the water again over him with the ſame happy effect. He had a good night's reſt; next morning he was free of fever, and he was diſmiſſed cured on the third day from his admiſſion.

"Pleaſed with this ſucceſs, I immediately adopted the ſame practice in nine other caſes of fever, from the firſt to the fifth day of the diſeaſe, with equal benefit. In ſix other caſes, from the ſixth to the tenth of the diſeaſe, I followed the ſame practice; in three of theſe with complete and nearly immediate ſucceſs, having uſed affuſion only thrice with each. From the debility however which had been induced by the longer continuance of the diſeaſe, they were not ſo immediately fit to be diſcharged from the hoſpital. In the three other caſes ſenſible relief was obtained, and they all recovered in the end, but the diſeaſe ran its courſe. Encouraged by the ſucceſs and ſafety of the practice, in one inſtance I went ſo far as to try the cold affuſion on the ſixteenth day of fever. At the moment, the patient ſeemed almoſt inſenſible to the ſhock. When he was replaced in bed, his extremities and his ſurface were cold; the pulſe became fluttering, and ſcarcely perceptible. We uſed frictions, and poured warm wine into him in ſmall quantities at a time, which he ſwallowed with great difficulty. He recovered his power of deglutition, and we increaſed the wine gradually to three bottles a day. He recovered in the end, but was upwards of three months in the hoſpital.

"In a few weeks, the old caſes that were on hand when I began the uſe of the cold affuſion were diſcharged, but the contagion, from the nature of the duty, continued to ſpread in the regiment, and many caſes of fever occurred in the months of Auguſt and September. Theſe were watched narrowly, the cold affuſion invariably uſed, and in general on the ſecond day. The effects were ſimilar to thoſe related of the caſe firſt mentioned; the ſucceſs was invariable. One of theſe caſes, that of Holding, of the grenadiers, was remarkable: He was taken into the hoſpital the 20th of September, late in the evening, of the ſecond day of fever, with the uſual ſymptoms, but in the ſevereſt form. An emetic was adminiſtered, and the affuſion deferred till morning. The emetic operated well, but his night was extremely reſtleſs, his head-ach particularly acute, and delirium came on with great violence. It was neceſſary to employ force to keep him in bed. In the morning of the 21ſt, (the third day of fever) I found his heat had ariſen to the uncommon height of 107°, and his pulfe was 125. The cold affufion was employed; he fcreamed from the feverity of the fhock; but, on returning into bed, he appeared much refrefhed, was perfectly fenfible, and faid that he thought himfelf well. On examining about ten minutes after the affufion, the heat was found to be 100°, the fkin moift, the pulfe 110. He flept for nearly four hours in perfect tranquillity. In the evening the fever returned. The affufion was repeated again with fimilar benefit. He flept the greater part of the night. Next morning the fever returned once more. The remedy was once more applied, and the difeafe was fubdued. He was difmiffed from the hofpital on the 29th.

"By this time the hofpital was thin from the rapid difcharge of the patients, but the contagion ftill continued to fpread in the regiment. The cold affufion was invariably applied, and, in general, on the fecond day of fever, as has already been mentioned. At length the contagion was extinguifhed; when we left Gofport, in the month of November, not a fingle cafe of fever was left behind. From the end of July to the 31ft of October, I employed the cold affufion in fixty-four cafes. In fixty of thefe I arrefted the difeafe, having feldom occafion to ufe the remedy more than twice or thrice, and in no one cafe more than four times. In the other four cafes, (all of which are alluded to in the courfe of this narrative) the difeafe being advanced, was not ftopped by the remedy, though the patients ultimately recovered.

"From the time I began the ufe of the cold affufion, I ufed little or no wine, no opium, nor, indeed, almoft any other remedy, in any one cafe in which the cold affufion was employed.

"A great part of thefe facts was witneffed by Dr. Franklin, phyfician to the diftrict; nearly the whole of them by my fellow-furgeons, Mr. Varenne and Mr. Worthington; and the general truth of this reprefentation will be confirmed by my friends, the officers of the Chefhire, particularly by Major Buckworth, who frequently vifited the hofpital. I have the pleafure of adding, that any evening on parade, I can point out the individuals whofe cafes I have mentioned.

I have the honour to be, your very obedient fervant,

JAMES MARSHALL, Surgeon, Royal Chefhire Militia."

Any comment that I could make on this narrative would only weaken its effects. Experiments fuch as thefe, fo clear and fimple, on fo large a fcale, and performed before fo many witneffes, cannot, it fhould feem, be invalidated. They correfpond entirely with my daily experience, which in this refpect refpect poffeffes an uniformity which I fhould look for in vain in almoft any other branch of medical practice. The reader will have the goodnefs to notice, that Mr. Marfhall's practice was in exact conformity with the directions in the "Medical Reports." It was the low contagious fever that his patients laboured under; it was in the early ftage of the difeafe that he employed the remedy, and generally in the ftate of the greateft heat and exacerbation; laftly, it was affufion, not immerfion, that he employed.

In fevers arifing from, or accompanied by, topical inflammation, my experience does not juftify the ufe of the cold affufion; though in a great variety of thefe cafes, the warm affufion may be ufed with advantage. And though I have ufed the cold affufion in fome inftances fo late as the twelfth and even fourteenth day of contagious fever, with fafety and fuccefs, yet it can only be employed, at this advanced period, in the inftances in which the heat keeps up fteadily above the natural ftandard, and the refpiration continues free. In fuch cafes I have feen it appeafe agitation and reftlefsnefs, diffipate delirium, and, as it were, fnatch the patient from impending diffolution. But it is in the early ftages of fever (let me again repeat) that it ought always to be employed, if poffible; and where, without any regard to the heat of the patient, it is had recourfe to in the laft ftage of fever, after every other remedy has failed, and the cafe appears defperate, (of which I have heard feveral inftances) can it appear furprifing that the iffue fhould fometimes be unfavourable?"

Some of my correfpondents complain of the difficulty of perfuading their patients to fubmit to the cold affufion. This difficulty will foon difappear, if, before the remedy is propofed, the practitioner fhall have firft decided in his own mind on its propriety; and in recommending it, fhall exhibit that calmnefs and confidence with which every remedy, and efpecially a new and an apparently bold one, fhould be propofed to a patient whofe mind is weakened by difeafe. Let me add, that every arrangement fhould be made for the affufion before the patient is moved at all, and that fatigue, as well as difquiet, fhould be particularly avoided. And, after all, the tepid affufion may be fubftituted for the more powerful remedy, in cafes where the delicacy of the fyftem, or the apprehenfions of the patient, or of the bye-ftanders, prevent the other from being employed.

Since the publication of the "Medical Reports," the cold and tepid affufion have been applied to fome other difeafes, and particularly to the cynanche maligna and fcarlatina anginofa, with the happieft effects. Of this I propofe to give fome account in a fubfequent Number of your Journal. It is to be lamented, lamented, that in the habitations of the poor there is ſeldom an opportunity of uſing theſe remedies ſufficiently early, or with the accommodations which are ſo deſirable towards enſuring comfort, ſafety, and ſucceſs. Liverpool, with its uſual public ſpirit, is erecting an extenſive Hoſpital for the reception of Fever and other contagious diſeaſes, which will, in ſome meaſure, remedy this and many other evils. The typhus and ſcarlet fever have of late been unuſually prevalent in Liverpool, and to theſe have been added a bowel complaint, aſſuming very generally the form of dyſentery. This has been in a very uncommon degree general and ſevere; but the report that the yellow fever has made its appearance among us is entirely without foundation. Of theſe epidemics ſome authentic particulars may in due ſeaſon be expected by the public. I have the honour to be, Dear Sir,

Your obedient ſervant,

